# Quantitative transmission ultrasound tomography: Imaging and performance characteristics

**DOI:** 10.1002/mp.12957

**Published:** 2018-05-28

**Authors:** Bilal Malik, Robin Terry, James Wiskin, Mark Lenox

**Affiliations:** ^1^ QT Ultrasound 3 Hamilton Landing, Suite 160 Novato CA 94949 USA

**Keywords:** imaging performance, inverse scattering, QT ultrasound, transmission ultrasound, ultrasound tomography

## Abstract

**Purpose:**

Quantitative Transmission (QT) ultrasound has shown promise as a breast imaging modality. This study characterizes the performance of the latest generation of QT ultrasound scanners: QT Scanner 2000.

**Methods:**

The scanner consists of a 2048‐element ultrasound receiver array for transmission imaging and three transceivers for reflection imaging. Custom fabricated phantoms were used to quantify the imaging performance parameters. The specific performance parameters that have been characterized are spatial resolution (as point spread function), linear measurement accuracy, contrast to noise ratio, and image uniformity, in both transmission and reflection imaging modalities.

**Results:**

The intrinsic in‐plane resolution was measured to be better than 1.5 mm and 1.0 mm for transmission and reflection modalities respectively. The linear measurement accuracy was measured to be, on average, approximately 1% for both the modalities. Speed of sound image uniformity and measurement accuracy were calculated to be 99.5% and <0.2% respectively. Contrast to noise ratio (CNR) measurements vary as a function of object size.

**Conclusions:**

The results show an improvement in the imaging performance of the system in comparison to earlier ultrasound tomography systems, which are applicable to clinical applications of the system, such as breast imaging.

## 1 Introduction

The idea of ultrasound tomography has been an object of research since the paper of Wild and Reid.^1^ Research interest was rekindled with the early papers of Johnson, Greenleaf and Bahn and coworkers at the Mayo Clinic.^2^, ^3^ Other early work included that at General Electric and the University of Colorado.^4^, ^5^ This was followed by research at the University of Utah resulting in several papers and dissertations from the Johnson Advanced Imaging Methods (AIM) Lab in the Department of Bioengineering at University of Utah.^6^, ^7^ Simultaneously research was carried out in other centers, including the Karmanos Research Center.^8^, ^9^

The modern era began with the pioneering work of Greenleaf and Johnson. At that time, simple straight‐ray tomography was used to create low resolution tomographic images of the human breast immersed in a water bath. Although these images of tissue characteristics were low resolution and crude by today's standards, they showed that quantitative information could be a valuable adjunct to standard hand‐held ultrasound (HHUS) — i.e. B‐mode images. Early work of Carson et al. also showed the importance of the quantitative measurement of breast tissue.^10^ The confounding factors in these images reconstructed using a 2D model were considered.^11^ The work of Li et al. utilized a bent‐ray tomographic algorithm to substantially improve the resolution of the image over the straight ray approximation.^12^ They have also done extensive work with bent‐ray tomographic method to image breast tissue *in vivo* as well as more recently the frequency domain approach similar to the methods used here.^12^, ^13^, ^14^ All but their most recent work in the frequency domain, however, are 2D in nature, due to the computational complexity involved in the inversion of the full Helmholtz equation.^14^

In addition, Ruiter et al. have used the bent‐ray model in 3D (not 2D) to further improve the image quality.^15^ This is to be expected since the true ray path in an inhomogeneous breast will indeed travel in a full 3D manner and will not be constrained to a single plane. In fact, it is known that even constraining an acoustic field that is passing through a homogeneous water bath is not possible due to diffraction effects.

The ray based methods, which have been successful in geophysics, are based on an infinite frequency assumption as they follow from the Eikonal equation, which in turn, is derived from an asymptotic expansion of the solution to the Helmholtz equation. Thus, they do not take into account diffraction effects and result in, therefore, poor resolution images. The forward scattering problem, as a function of the scattering potential, is a nonlinear problem by virtue of the implicit dependence of the scattered field on the scattering potential. There are, however, linear approximations to the forward and hence inverse problem. Indeed, when ray‐tracing is used, Fermat's principle states that the ray paths are, to second order, stationary with respect to variation in the scattering potential. This reflects the fact that the ray tracing solution does not include diffraction effects which are responsible for high resolution in the images.

On the other hand, linear approximations to the forward scattering problem in the Lippmann‐Schwinger equation lead to the Born or Rytov approximations. These have been investigated by Lavarello et al. in 2D and 3D, and others.^16^, ^17^ Unfortunately, these methods fail for the contrast and size of the human breast.^18^, ^19^ Attempts to carry these ideas to higher order, the so‐called “distorted wave Born inversions”, have also not been as successful as required for clinical use.

Wiskin et al. derived methods based on the parabolic approximation to the Helmholtz equation which provide 3D quantitative estimates of tissue characteristics at the millimeter scale.^19^, ^20^ Furthermore, the 3D speed and attenuation maps produced are used to correct for refraction and gain in a refraction corrected reflection algorithm. The resulting reflection image is essentially a refraction‐corrected 360‐degree compounded B‐scan. The three tissue characteristic based images so obtained are of sufficient quality that they have been utilized in a Support Vector Machine (SVM) classifier to classify breast tissue as skin, fat, glands, ducts, or connective tissue with an overall accuracy of over 90%.^21^

Quantitative Transmission (QT) Ultrasound is an FDA approved breast imaging modality approved for use as an adjunct to mammography. QT does not require compression, so it is comfortable and fast, requiring only a few minutes for a typical scan. It is substantially different from hand‐held ultrasound (HHUS) in that it is a fully automated 3D modality. No direct operator interaction is required to generate 3‐dimensional image maps of breast tissue. There are no industry consensus standards for determination of performance of QT systems and FDA guidance for ultrasound is not designed around the concept of a tomographic multimodality device. A previous paper made an initial attempt to reconcile these issues.^22^ That initial work combined with recent interaction with the FDA performed for the purposes of 510(k) approval has resulted in the development of a series of performance tests that are comprehensive and can form the basis for future regulatory guidance as well as industry standards. In this new series of tests, we evaluate both accuracy and precision of the system to measure quantitative values as well as spatial information. These measurements are performed in all three directions as this is a 3D imaging modality. In addition, more typical tomographic measurements like uniformity, contrast‐to‐noise, and spatial resolution/point‐spread‐function (PSF) are also measured for both transmission and reflection modalities.

## 2 Materials and methods

### 2.A. System description

**Figure 1:**
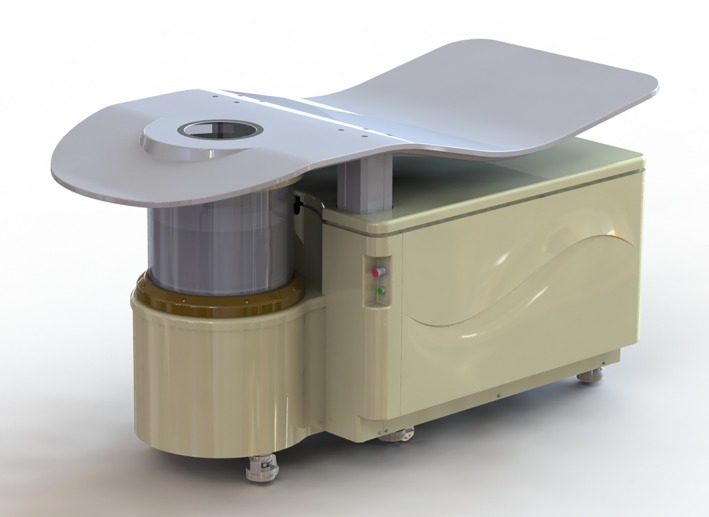
3D CAD rendering of the QT Scanner 2000. [Color figure can be viewed at http://wileyonlinelibrary.com]

We evaluated the performance characteristics of the newer generation QT Ultrasound system: QT Scanner 2000. While similar to older generation QT systems, a major difference is the use of a 2048‐element receiver instead of a 1536‐element receiver in the older systems, and correspondingly the size of each individual transducer element is 0.5 mm in the newer generation in comparison to 0.65 mm in the older generation — i.e. a 30% improvement in both size and number of elements.^22^ Also, the field of view is wider by approximately 20 mm in the new design to accommodate larger patients. In addition, further enhancements have been made for patient comfort and ergonomics. A rendering of the new design is shown in Fig. 1.

**Figure 2:**
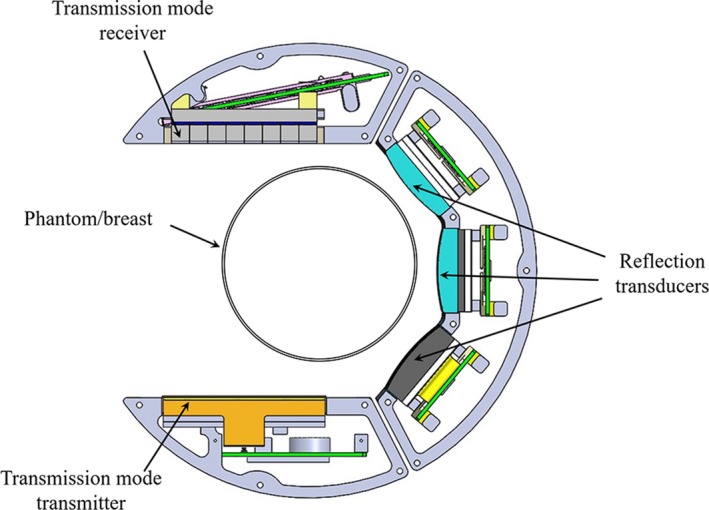
Schematic of the scan head within the QT scanner. At each level, the scan head performs a full 360‐degree rotation before stepping to the next level. Within each level, the transmitter‐receiver array combination (transmission mode) and the reflection arrays time multiplex the acquisition. The three reflection transducers are of different focal length such that their depth of foci combined with rotation/translation of the scan head covers the full imaging volume. Note that there is an offset between the center of the breast and the center of the transmission arrays. The 360‐degree rotation insures that all parts of the breast, at some point, are in between the transmitter‐receiver pair and that there is indeed complete coverage of k‐space (i.e., spatial Fourier transform space), as can be most easily seen with an Ewald circle analysis. [Color figure can be viewed at http://wileyonlinelibrary.com]

A description of the hardware is provided elsewhere.^22^ A schematic of the scan head is shown in Fig. 2. In the transmission mode, the transmitter emits a wave that is approximately constant in phase in the direction parallel to the longest dimension of the transmit array. In the direction perpendicular to this, i.e. in the vertical direction with reference to the water tank and array, the signal is focused to a distance of 60 mm. Thus, it is an approximation to a plane wave in a finite space. As our inversion is model based, as long as we model this incident field accurately, it does not have to be a perfect plane wave. The wave traverses the scan tank and is received at the 2048‐element array. This receiver consists of eight rows and 256 columns of array elements, in blocks of 32 columns. The pitch in the horizontal direction is 0.5 mm, and the pitch in the vertical direction is 2.5 mm. Thus, the array is 128 mm wide and 20 mm tall. A wideband chirp provides raw information at frequencies ranging from 0.3 to 1.5 MHz, with a center frequency for the transmission transmitter/receiver pair at ~0.9 MHz and 6‐dB bandwidth of approximately 1 MHz and is acquired for 180 angles as the set of arrays is rotated around the object. The acquired projections are used for image reconstruction using nonlinear inverse scattering in 3D, result of which is three‐dimensional image volumes of complex refractive index values — the real and imaginary parts of the refractive index correspond to the speed of sound and attenuation.^19^, ^20^, ^23^ Note that the system is able to collect out of plane information since the receiver array is 20 mm tall and consists of eight rows of receiver elements. Furthermore, even though the receiver array only samples a subset of the vertical extent of the field, the backpropagation and forward simulation algorithms are fully 3D. This means that one level of data will, in fact, affect a large number of the image space levels. Conversely, all of the image levels are affected by all of the data levels, at least, insofar as the field generated by our transmitter has vertical extent.

The reflection arrays have a center frequency of 3.6 MHz with ~70% bandwidth (6‐dB down) — according to manufacturer specifications. The individual arrays consist of a single row of 192 elements with a pitch of 0.375 mm. These are vertically focused at 25, 45, and 75 mm, and have respective elevational heights of 4, 7.5, and 12 mm. These reflection arrays are dynamically focused with beamformers, in the horizontal direction. The choice of the transducer bandwidth is dictated by considerations of depth of penetration and throughput. The attenuation of the breast varies from breast to breast depending on percentage of fibroglandular tissue and other characteristics. The lateral resolution from theoretical considerations is estimated to be approximately $\lambda /\sqrt{2}$, based on an Ewald circle analysis. Hence, the desire for high frequencies is counterbalanced by the increased attenuation at these frequencies. Our choice is a compromise between these conflicting requirements. A single view is comprised of contributions from each of the three reflection arrays (short‐, medium‐, and long‐ range foci). The acquired multiple views are spatially compounded which result in significantly reduced speckle. These reflection images are also corrected for refraction using the corresponding speed of sound images. The outcome is a 3D volume of essentially three different modalities: speed, attenuation, and reflection.

In this study, we focus on characterizing the speed of sound and reflection images. Nevertheless, the attenuation image is used in a coarse way to correct for gain in the reflection image. This correction is based on the total size of the breast as determined from the attenuation image. This is a gross correction precisely because of the coarse nature of attenuation images at this stage of development.

### 2.B. Performance characteristics and fabrication of phantoms

We designed and evaluated multiple phantoms to quantify the performance characteristics of the QT imaging system. There were two materials used for phantoms: agar phantoms fabricated in house, and polyurethane phantoms fabricated by CTi (Conversion Technology, Inc., Boulder, CO). The phantoms’ design specifications (size of the phantoms, size of the inclusions, and speed of sound of background and inclusions) were provided by QT Ultrasound to CTi who fabricated the phantoms, followed by testing and characterization at QT Ultrasound. The speed values for each phantom were tested independently using two collinear ultrasound piston transducers (V323‐SM, Olympus America, Inc., Waltham, MA, USA). The experimental setup included: two pistons at a set distance apart using an absolute digital readout (with sub‐100 μm accuracy), phantom placed between the two pistons with phantom's sides coupled flat with the pistons, and a temperature‐controlled water bath. The speed of sound characterization of the phantoms was performed by measuring the time of flight in the phantom in comparison to that in water. The procedure was repeated for all the phantoms. Once the design specifications were finalized, the phantoms were scanned in the QT Scanner 2000. After scanning and image reconstruction, the measurements were performed as detailed below.

#### 2.B.1. Spatial resolution

##### Speed of sound phantom

**Figure 3:**
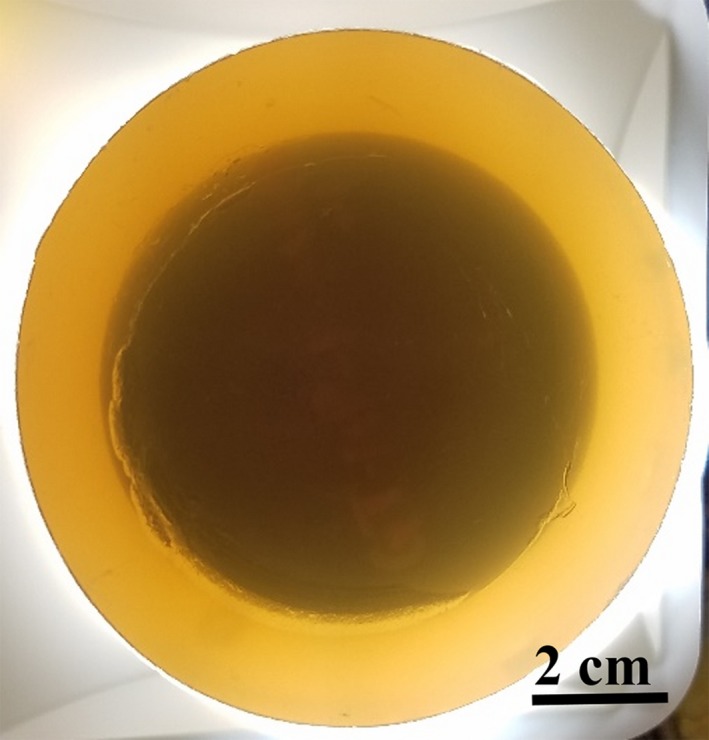
Speed of sound resolution phantom: a high‐speed polyurethane phantom can be seen embedded within the agar gel. [Color figure can be viewed at http://wileyonlinelibrary.com]

We fabricated a cylindrical agar phantom with a smaller cylindrical urethane phantom embedded within. The urethane medium was of relatively higher and uniform speed of sound (1566 m/s) in comparison to background agar gel (1517 m/s). The 3% agar gel was prepared by dissolving 15 g of agar (select agar, Sigma‐Aldrich) in 500 ml of deionized (DI) water. The temperature of the solution was raised to over 85 degrees Celsius under constant stirring to completely dissolve the agar in water. The solution was then degassed and poured into the mold. High speed urethane phantom was then embedded within the phantom. A photo of the phantom is shown in Fig. 3.

##### Reflection phantom

A two‐layer cylindrical agar phantom with glass beads as inclusions was fabricated. The 3% agar gel was prepared by dissolving 21 g of agar in 700 ml of DI water. The temperature of the solution was raised to over 85 degrees Celsius under constant stirring to completely dissolve the agar in water. The solution was then degassed and approximately half of it was poured into the mold. Three 100‐μm soda lime glass beads (Cospheric, LLC, Santa Barbara, CA) were used to measure the reflection measurement resolution. These beads were scattered sparsely on top of the poured agar. The remaining agar solution was then poured on from the top, encapsulating the beads within the two agar layers.

#### 2.B.2. Linear measurement accuracy

##### Speed of sound phantom

**Figure 4:**
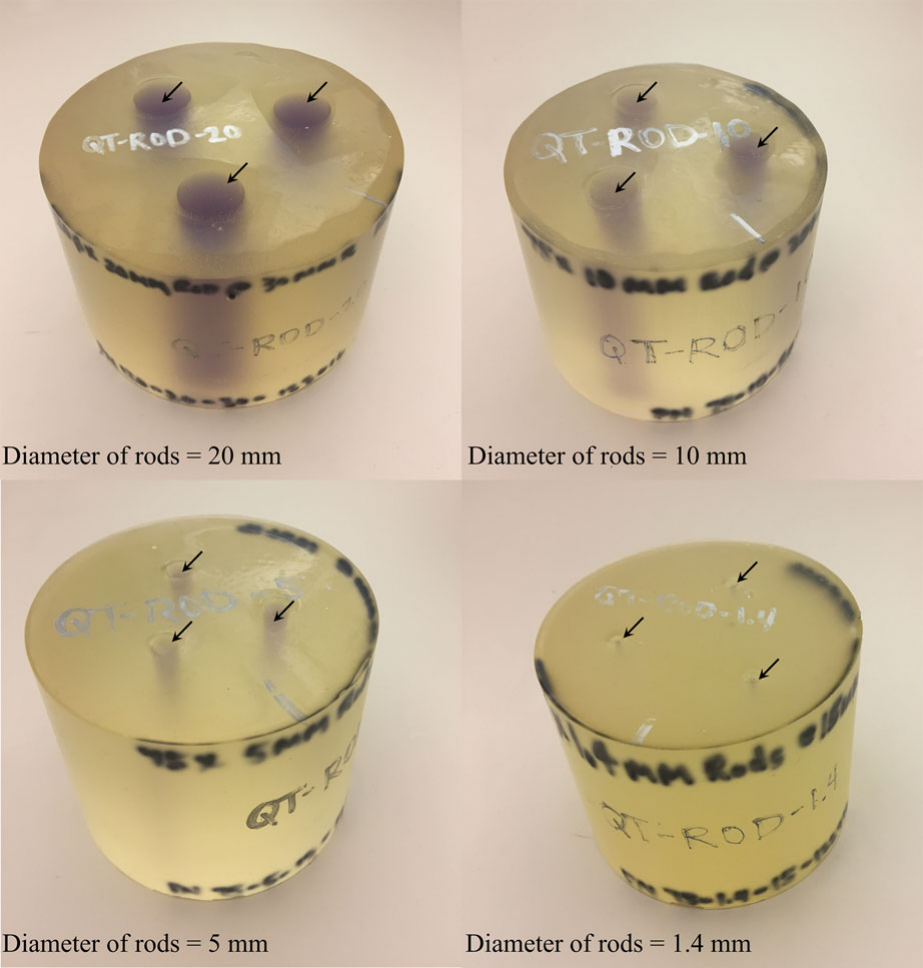
Urethane phantoms used for measurement of linear measurement accuracy and contrast to noise ratio in QT speed of sound imaging. The arrows mark the location of the inclusions. [Color figure can be viewed at http://wileyonlinelibrary.com]

Linear measurement accuracy is defined as the accuracy of performing linear distance measurements within an image slice that is, distance from one point to another within an image. We used three different breast tissue urethane phantoms representative of breast parenchyma embedded in fat tissue. The phantoms were cylindrical shape with rod‐like inclusions. The inclusions were of diameter 5, 10, and 20 mm, with reference speed of sound of 1529.0 m/s. The reference speed of sound of the background urethane material was 1432.8 m/s. The speed of sound characterization in the phantoms was performed by measuring the time of flight in the phantom in comparison to that in water, as described above in Section 2.B. The phantoms were then scanned in the QT Scanner 2000 and a variety of dimensions were measured within these phantoms. These measurements included, for each of the viewing planes, the diameter of the cylindrical inclusions as well as the overall diameter and height of the phantoms. A photo of these phantoms is shown in Fig. 4.

##### Reflection phantom

A two‐layer agar phantom with glass beads was created to quantify the linear measurement accuracy in QT reflection imaging. The 3% agar gel was prepared by dissolving 21 g of agar in 700 ml of DI water, as described above. Several 0.7 mm glass beads were spread out between the two layers of the agar phantom. The phantom was then scanned in three orthogonal positions within the scan tank to allow measurement of the same distance within the coronal plane as well as in axial and sagittal planes. While the dimensions of the phantom were measured using a caliper, the dimensions/distances in between the glass beads within the phantom were measured using a calibrated X‐ray image (on GE Senographe Essential).

#### 2.B.3. Image uniformity

##### Speed of sound phantom

Image uniformity is defined as signal pixel intensity uniformity within an image that is repeatable scan to scan. Cylindrical puck‐shaped phantoms of homogeneous composition were used to determine the image uniformity in QT speed imaging. A stack of such phantoms, with each puck of different speed of sounds value, was scanned in the QT scanner and the speed of sound images were analyzed by drawing a region of interest (ROI) within the phantom image. The same phantoms were also used to measure the speed of sound accuracy of the QT system.

We used NEMA's (National Electrical Manufacturers Association) definition of measurement ROI which is defined as a centered, regular geometric area enclosing at least 75% (area) of the image of the signal producing region of the phantom.^24^ The uniformity was calculated as normalized absolute average deviation (NAAD) which is essentially the complement of standard deviation calculated across the ROI divided by the mean of the ROI. NAAD is computed as:

$$NAAD=100\left(1-\frac{1}{\mathrm{NY}}\sum_{i=1}^{N}\left(\left|Y_{i}-\overset{¯}{Y}\right|\right)\right)$$

where $Y_{i}$ is the individual pixel value in the ROI, $\overset{¯}{Y}$ is the mean of all pixels in the ROI, and *N* is the total number of pixels in the ROI.

##### Reflection phantom

Since reflection is a measure of impedance mismatch, uniformity for reflection images is not well‐defined. For instance, a truly uniform phantom would appear anechoic and hence would have a mean pixel value of zero, thereby, making the uniformity calculation unstable. Further work is needed to characterize the reflection uniformity.

#### 2.B.4. Contrast to noise ratio

##### Speed of sound phantom

In addition to the phantoms described in Section 2.B.2, a similar phantom with cylindrical rods of 1.4 mm was also used for analysis. Contrast to noise ratio (CNR) is a measure of the potential to detect an object against a background that contains a noise component. For the current objective, we define CNR as the difference between a region containing the object and the immediate background, divided by the standard deviation of the overall background:

$$\text{CNR}=\frac{S_{\mathrm{obj}}-S_{\mathrm{bkd}}}{\delta_{\mathrm{bkd}}}$$

where S_obj_ is the signal from the object, S_bkd_ is the signal from the area around the object, and *δ*_bkd_ is the standard deviation across the overall background. For any given object, an ROI of diameter just under the size of the object was drawn on the object and the mean value of intensity within that ROI was used as the signal from the object. Another ROI was drawn within the urethane embedding medium which served as the signal from the immediate background of the object. The standard deviation across the image of surrounding water was used as the noise floor.

##### Reflection phantom

The method to analyze CNR in reflection mode is as described above for that of speed of sound imaging. Instead of high speed of sound inclusions, soda lime glass beads (Cospheric LLC) of size 1000, 800, 550, 300, 200, and 100 μm were used as high reflection objects within agar background.

### 2.C. Implementation

The methods described above were implemented in MATLAB (R2017a, Mathworks, Natick, MA) and ImageJ (National Institutes of Health, Bethesda, MD) on a standard computer workstation (Intel Core i7 3.6 GHz, 16 GB RAM). Both custom written routines and built‐in functions and plugins were used.

## 3 Results and discussion

### 3.A. Spatial resolution

#### 3.A.1. Speed of sound imaging

**Figure 5:**
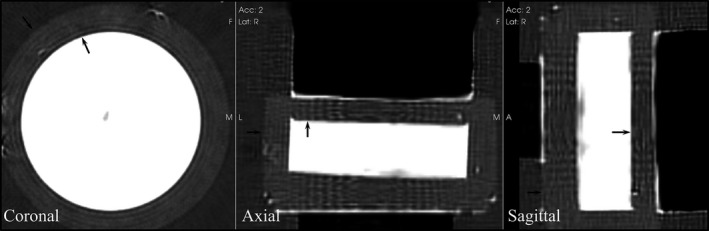
QT speed image of the speed of sound resolution phantom. The phantom (white) is embedded within agar. The black arrows mark the interface between the phantom and the agar gel. The white arrows mark the interface between agar gel and water. The black region in the axial and sagittal views is material to support the phantom within the scan tank.

The QT speed image of the speed‐of‐sound resolution phantom is shown in Fig. 5. Note that the black arrows mark the interface between the urethane and agar gel, and the white arrows mark interface between the agar gel and the surrounding water. The black region seen in the axial and sagittal views corresponds to supporting materials used to hold the phantom in the scan tank.

**Figure 6:**
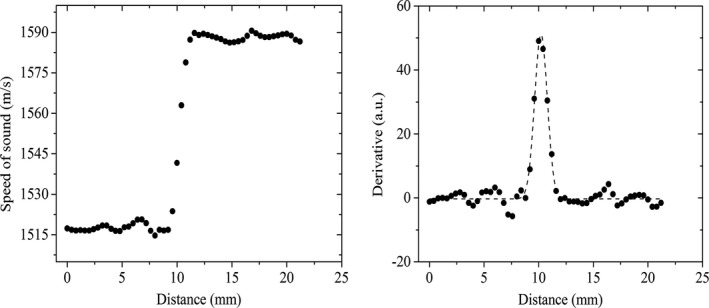
(Left) Data points from an example line profile across the urethane‐agar interface. The line profile represents the edge‐spread function across the interface; (Right) First derivative of the edge‐spread function with the corresponding Gaussian function fit (dashed line). The Gaussian fit represents the point spread function and the respective FWHM is the resolution.

The resolution was measured by calculating the derivative of the edge spread function. Line profiles drawn across the interface of urethane and agar gel served as the edge spread functions. The data points (black squares) for a representative edge spread function in the coronal plane are shown in Fig. 6. This was followed by taking the first derivative of this edge spread function. The derivative of the line profile was fitted to a Gaussian distribution and the corresponding full‐width‐half‐max (FWHM) was measured. The measurements were performed three times each in all three viewing planes, and both the average and standard deviation values were calculated. The mean values of the resolution and respective precision are provided in the Table 1 below. The precision values were reported as one standard deviation across multiple (n = 3) measurements.

**Table 1:** FWHM of the point‐spread function measured in QT speed of sound imaging. X and Y axes correspond to the coronal view plane (lateral direction). Z axis corresponds to the axial and sagittal planes (axial direction)

Position	FWHM (mm)
X and Y	1.49 ± 0.07
Z	2.35 ± 0.11

#### 3.A.2. Reflection imaging

**Figure 7:**
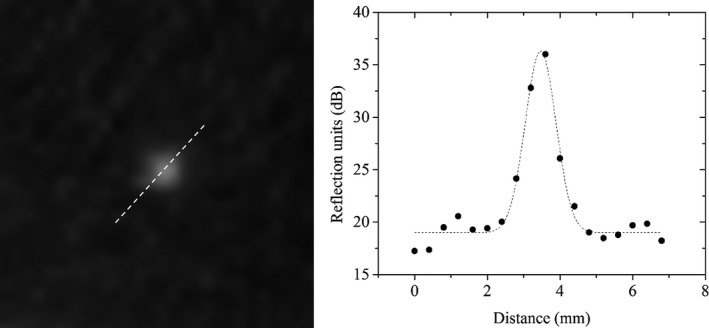
Resolution measurement in reflection imaging: the data points are the actual intensity values of the line profile across the images of glass beads. The dashed line is the corresponding Gaussian curve fitting and represents the point spread function.

A section of the QT image of the reflection resolution phantom is shown in Fig. 7 with the respective intensity line profiles. The resolution was calculated by measuring the FWHM of the intensity line profiles in both lateral and axial directions. The mean values of the resolution and respective precision are provided in the Table 2 below. The precision values are reported as one standard deviation across multiple (n = 3) measurements.

**Table 2:** FWHM of the point‐spread function measured in QT reflection imaging. X and Y axes correspond to the coronal view plane (lateral direction). Z axis corresponds to the axial and sagittal planes (axial direction)

Position	FWHM (mm)
X and Y	0.96 ± 0.11
Z	3.19 ± 0.32

### 3.B. Spatial measurement accuracy

#### 3.B.1. Speed of sound imaging

**Figure 8:**
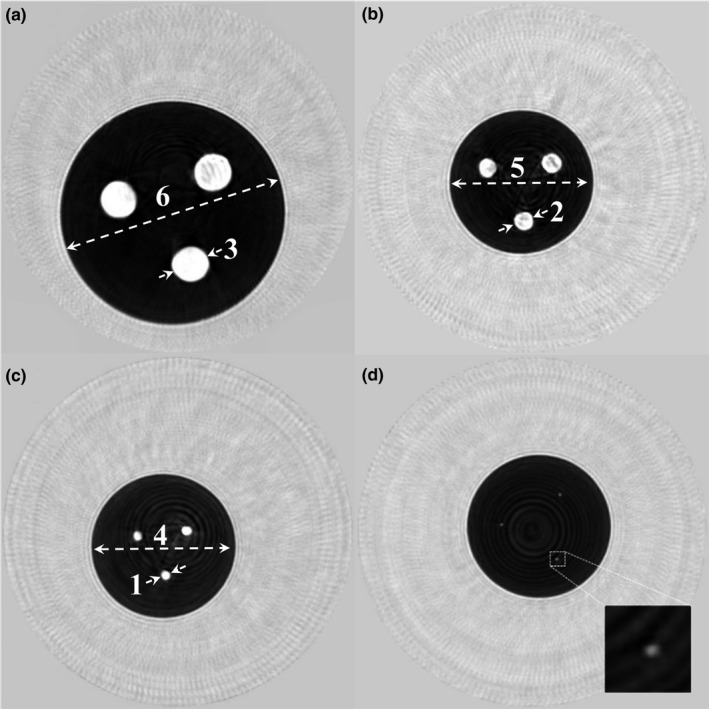
Coronal view speed of sound images of cylindrical phantoms shown in Fig. 4. The four phantoms have cylindrical rods of diameter (a) 20 mm, (b) 10 mm, (c) 5 mm, and (d) 1.4 mm. The rods can be clearly seen as bright (higher speed of sound) circular regions within a darker (lower speed of sound) background medium. The numbers next to dashed lines correspond to “Measurement #” column in Table 3.

The phantoms shown in Fig. 4 were scanned in the QT Scanner 2000 and the corresponding speed of sound images are shown in Fig. 8. Note that the higher speed cylindrical rods can be clearly seen within the darker background.

The results of these measurements are summarized in Table 3. Note that the bias values are calculated as difference between the actual and measured values and hence represent the accuracy of measurement. The precision values were calculated as both one standard deviation across the multiple measurements (n = 3), and as coefficient of variation which is the ratio of standard deviation and mean value.

**Table 3:** Linear measurement accuracy and precision in QT speed of sound imaging. Note that the bias values represent the measurement accuracy calculated as difference between actual values (measured by calipers) and the value measured by QT, and the percentage bias values are calculated as percentage ratio of bias and actual values. The “Measurement #” column identifies the numbered measurements in Fig. 8

Imaging plane	Measurement #	Measured by calipers (mm)	Measured QT (mm ± SD) (n = 3)	% SD (coefficient of variation) (n = 3)	Bias (mm)	% bias
Coronal	1	5.00	5.00 ± 0.17	3.46	0.00	0.00
	2	10.00	10.07 ± 0.06	0.57	+0.07	0.66
	3	20.00	20.53 ± 0.75	3.66	+0.53	2.63
	4	75.60	76.50 ± 0.26	0.35	+0.90	1.18
	5	76.10	76.77 ± 0.50	0.66	+0.67	0.87
	6	119.70	119.23 ± 0.40	0.34	−0.47	0.39
			Mean coefficient of variation = 1.51		Mean % difference: = 0.96	
Axial	1	5.00	5.13 ± 0.06	1.12	+0.13	2.63
	2	10.00	10.10 ± 0.40	3.96	+0.10	1.00
	3	20.00	19.87 ± 0.42	2.10	−0.13	0.67
	4	60.10	60.00 ± 0.66	1.09	−0.10	0.17
	5	60.10	59.53 ± 0.21	0.35	−0.57	0.95
	6	76.50	75.23 ± 0.40	0.54	−1.27	1.67
			Mean coefficient of variation = 1.53		Mean % difference = 1.18	
Sagittal	1	5.00	5.17 ± 0.06	1.12	+0.17	3.28
	2	10.00	10.07 ± 0.06	0.57	+0.07	0.66
	3	20.00	20.10 ± 0.10	0.50	+0.10	0.50
	4	60.10	59.20 ± 0.20	0.34	−0.90	1.51
	5	60.10	59.47 ± 0.32	0.54	−0.63	1.06
	6	76.50	76.33 ± 0.65	0.85	−0.17	0.22
			Mean coefficient of variation = 0.65		Mean % difference: = 1.20	

#### 3.B.2. Reflection imaging

**Figure 9:**
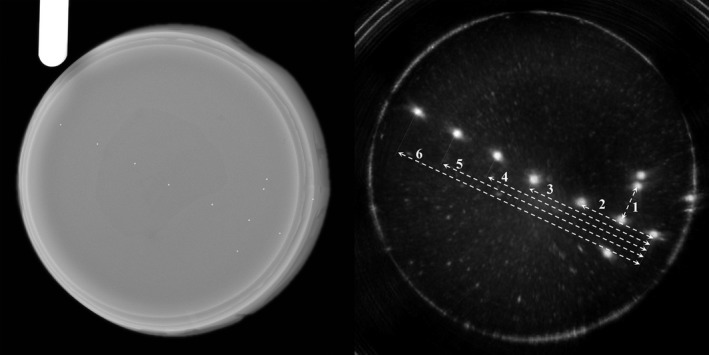
X‐ray image (left) and the QT reflection (right) of the reflection linear accuracy phantom. The glass beads seen in the X‐ray image can be clearly seen in the QT reflection image. The white arrows mark the dimensions that were measured to determine the linear measurement accuracy. The numbers next to dashed lines correspond to “Measurement #” column in Table 4.

The QT reflection image and the corresponding X‐ray image are shown in Fig. 9. Due to the cone‐beam nature of X‐ray, the image is slightly magnified. Therefore, we used a gauge of known length (32 mm) in order to compensate for that magnification, before comparing the measurements in X‐ray and QT images. Multiple measurements (n = 3) were performed on the same beads in coronal, axial, and sagittal views, and the respective accuracy and precision values were calculated. The results are summarized in Table 4.

**Table 4:** Linear measurement accuracy and precision in QT reflection imaging. Note, the bias values represent the measurement accuracy calculated as difference between actual values (measured by x‐ray) and the value measured by QT, and the percentage bias values are calculated as percentage ratio of bias and actual values. The “Measurement #” column identifies the numbered measurements in Fig. 9

Imaging plane	Measurement #	Measured by X‐ray (mm)	Measured QT (mm ± SD) (n = 5)	% SD (coefficient of variation) (n = 3)	Bias (mm)	% bias
Coronal	1	13.82	13.65 ± 0.17	1.28	−0.17	1.21
	2	30.39	30.59 ± 0.18	0.59	+0.13	0.44
	3	50.18	50.36 ± 0.14	0.28	+0.18	0.36
	4	66.52	66.78 ± 0.18	0.26	+0.26	0.38
	5	83.99	84.37 ± 0.18	0.22	+0.38	0.45
	6	101.19	101.65 ± 0.19	0.19	+0.46	0.45
			Mean coefficient of variation = 0.46		Mean % difference = 0.55	
Axial	1	13.82	13.56 ± 0.16	1.18	−0.26	1.87
	2	30.39	30.17 ± 0.36	1.21	+0.22	0.73
	3	50.18	50.16 ± 0.30	0.60	−0.02	0.04
	4	66.52	66.49 ± 0.59	0.89	−0.04	0.06
	5	83.99	84.41 ± 0.66	0.78	+0.42	0.50
	6	101.19	100.83 ± 0.69	0.68	−0.36	0.35
			Mean coefficient of variation = 0.89		Mean % difference = 0.59	
Sagittal	1	13.82	13.43 ± 0.34	2.52	−0.39	2.89
	2	30.39	29.92 ± 0.39	1.30	−0.47	1.55
	3	50.18	49.71 ± 0.31	0.63	−0.47	0.94
	4	66.52	66.26 ± 0.57	0.86	−0.26	0.40
	5	83.99	83.91 ± 0.06	0.08	−0.08	0.10
	6	101.19	100.61 ± 0.11	0.11	−0.58	0.57
			Mean coefficient of variation = 0.92		Mean % difference = 1.07	

The absolute bias values in both speed of sound and reflection modalities are subresolution. Also note that there is no trend in the value of measurement and accuracy of measurement, which is a hallmark of true model‐based inversion tomographic techniques.^20^

### 3.C. Image uniformity

#### 3.C.1. Speed of sound imaging

**Figure 10:**
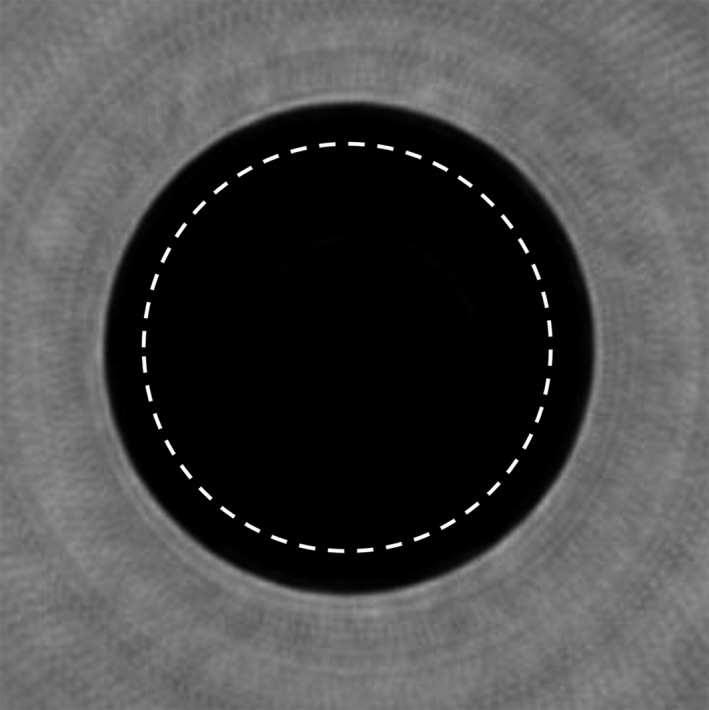
Coronal view of a uniform phantom. The dashed line represents the ROI across which the uniformity was calculated.

A representative coronal view image of one of the uniform phantoms is shown in Fig. 10. The NAAD uniformity was calculated for each puck phantom at multiple slices (n = 3), and the results are summarized in Table 5. The overall mean uniformity was calculated to be greater than 99%. The overall speed measurement accuracy (measured as bias from the reference speed value) was 0.17% with a precision of 0.16. Note that while bias values (in m/s) are calculated as difference between the actual and measured values, the percentage bias values correspond to the magnitude of the ratio of bias and actual value. The coefficient of variation is a measure precision and is calculated as the ratio of the standard deviation and the mean value. We would like to point out that the measure of uniformity employed here will give a slightly larger (better) value for uniformity in large regions of relatively slowly varying speeds of sound. Regions which have objects within them will have ROIs that have smaller measures of uniformity — even when the ROI is chosen to be a region of constant speed of sound. This is partially due to the nonlinear nature of our inversion/imaging process. Nevertheless, the measurement of the NAAD in large constant speed of sound regions is indeed optimistic, although certainly valid. It was done in line with NEMA methods which include selecting the ROI at the center of the image that includes 75% of the signal producing region.^21^

**Table 5:** Summary of speed of sound accuracy and uniformity measurements. The measurements were performed for five phantoms. The mean value is the average of three ROI measurements (n = 3) within each phantom. The SD values represent one standard deviation across all the number of pixels within an ROI. Uniformity is calculated as NAAD as described above and is essentially related to the percentage ratio of the standard deviation and mean values. Actual values are the reference speed of sound values for the phantoms as measured by the piston transducer setup. Bias values represent the speed measurement accuracy and are calculated as difference between the actual and the measured mean value. Percentage bias values are ratio of bias and actual speed of sound value. The coefficient of variation is a measure of speed of sound precision and is a ratio of SD (n = 3) and mean (n = 3)

Phantom#	Mean (m/s)	SD (m/s)	Uniformity (%)	Actual (m/s)	Bias (m/s)	Bias (%)	Coefficient of variation (%)
1	1515.5	6.4	99.58	1510	−5.5	0.36	0.22
2	1518.5	6.8	99.55	1516	−2.5	0.16	0.10
3	1432.0	5.1	99.64	1435	3.0	0.21	0.09
4	1493.5	7.8	99.48	1494	0.5	0.04	0.21
5	1449.2	5.9	99.59	1448	−1.2	0.08	0.17
		Mean=	99.57		Mean=	0.17	0.16

### 3.D. Contrast to noise ratio

#### 3.D.1. Speed of sound imaging

The image corresponding to CNR phantoms is shown in Fig. 8. The contrast to noise ratio was calculated as described above in Section 2.B.4. The ROIs were drawn within the area of the rods (n = 3). The measured CNR as a function of object size is summarized in Table 6. As expected, the CNR decreases as a function of the object size. The effect is more significant at object size of 1.4 mm which is on the order of the resolution of our speed of sound images. The CNR of smaller objects is also affected considerably by partial volume effects.

**Table 6:** QT speed of sound and contrast to noise (CNR) measurements as a function of object size. Mean values are average of n = 3 measurements. SD values are one standard deviation across the three measurements

Object size (mm)	Mean ROI speed (m/s)	Speed of sound CNR mean ± SD	Speed of sound CNR mean ± SD (dB)
20	1531.6	54.9 ± 0.70	17.4 ± 0.06
10	1527.2	53.8 ± 1.20	17.3 ± 0.10
5	1529.3	55.2 ± 0.58	17.4 ± 0.04
1.4	1497.5	36.9 ± 2.17	15.6 ± 0.26

#### 3.D.2. Reflection imaging

**Figure 11:**
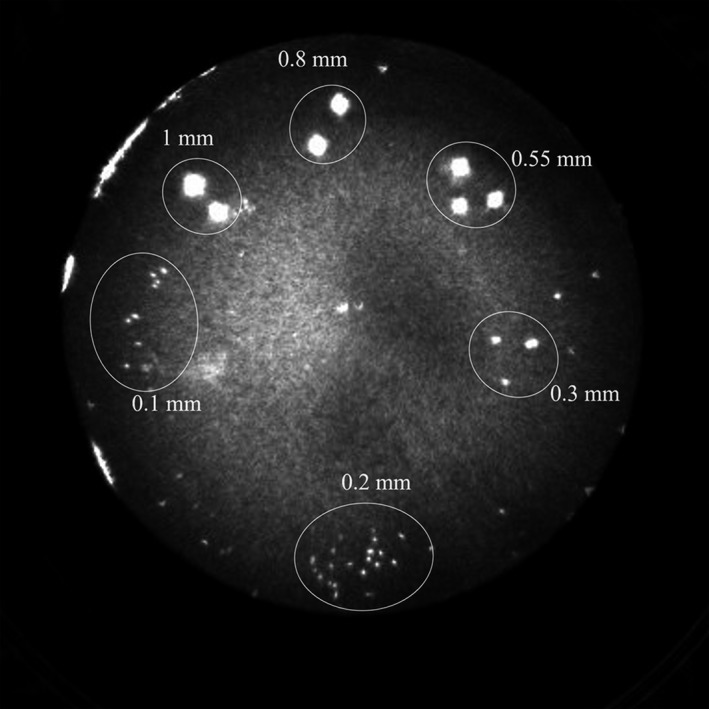
QT reflection image of the contrast to noise ratio (CNR) phantom. The glass beads of various sizes can be seen pooled together in subgroups.

The CNR in QT reflection was calculated using the same method as that of speed of sound. The refection imaging of the respective phantom is shown in Fig. 11. The ROIs were drawn within the area of the different sized beads and the respective CNR are summarized in Table 7. The reflection CNR stays relatively constant for sizes ranging from 1 mm to 0.3 mm and then decreases for smaller sizes. Unlike CNR for speed of sound, reflection CNR stays consistent for sizes much smaller than the resolution. This is due to the fact that the “detection” ability of QT reflection imaging goes beyond the ability to resolve between two objects, and unlike speed imaging, allows detection and visualization of subresolution objects.

**Table 7:** QT reflection and contrast to noise (CNR) measurements as a function of object size

Object size (mm)	Mean ROI intensity (RU)	Reflection CNR mean ± SD	Reflection CNR mean ± SD (dB)
1	1359800	2261.9 ± 350.4	33.5 ± 0.6
0.8	1111377	1825.1 ± 396.0	32.6 ± 0.9
0.55	1085997	1780.5 ± 218.1	32.5 ± 0.5
0.3	801039	1279.4 ± 149.6	31.1 ± 0.5
0.2	258373	325.1 ± 27.3	25.1 ± 0.4
0.1	189697	204.3 ± 11.6	23.1 ± 0.2

The performance metrics analyzed in this study show that the newer generation QT scanner (with 2048‐element receiver) shows significant improvement over our earlier generation scanner.^22^ Specifically, CNR for speed of sound imaging shows an improvement of ~3 dB (when comparing the 1.4 mm object). The transmission imaging spatial resolution also showed improvement — the new system shows lateral resolution of ~1.5 mm in comparison to >2 mm for the older generation scanner. A similar value of spatial resolution has been reported by Sandhu et al. for the SoftVue scanner.^14^ Image uniformity for the newer generation scanner was similar to that of the older generation, which is more dependent on our inversion algorithm than the number of receiver elements.

**Figure 12:**
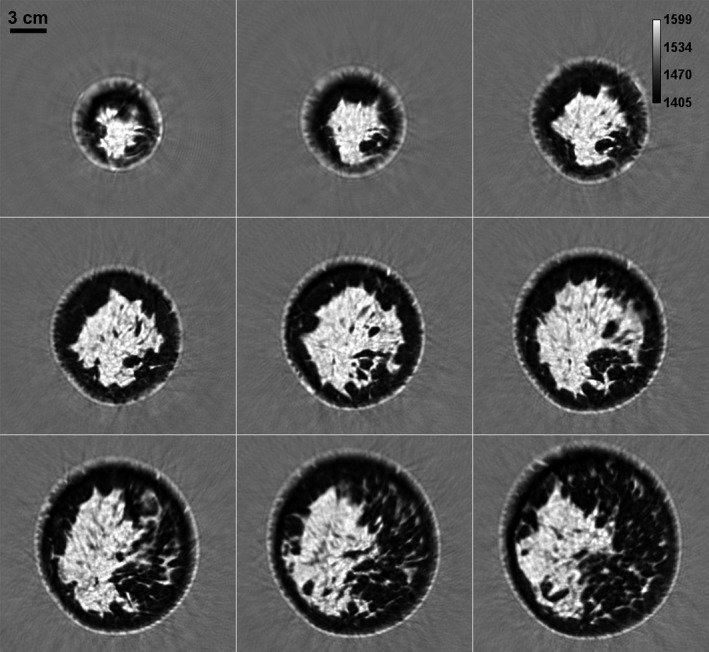
Speed of sound image montage from a whole breast *in vivo*. While the distance between two consecutive slices is 1 mm in the coronal view plane, the consecutive images (left to right and top to bottom) shown above differ by 5 mm separation. The scattered brighter region within the breast represents the fibroglandular tissue embedded within the darker fat tissue. The area surrounding the breast constitutes water in the scan tank.

**Figure 13:**
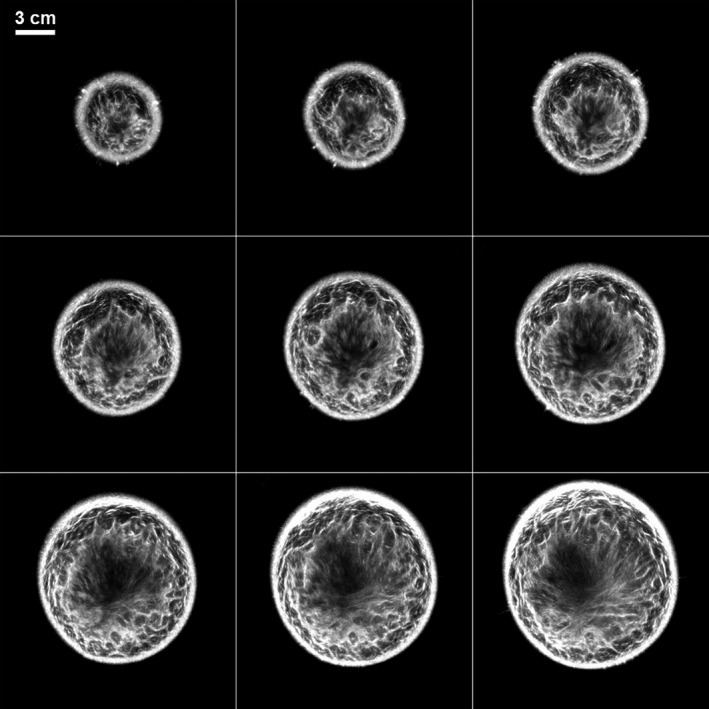
QT reflection images corresponding to the montage in Fig. 12. Note that the anatomy information provided in the reflection images is highly complementary to that in speed of sound images.

**Figure 14:**
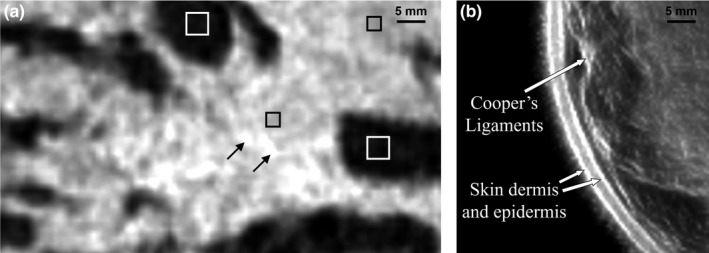
(a) Zoom‐in view of a QT speed of sound image of breast tissue: the white squares enclosing the dark region depict fat tissue; black squares mark the regions within the glandular tissue; black arrows mark the terminal ducto‐lobular units (TDLUs). (b) QT reflection image demonstrates delineation of high reflection interfaces such as multiple skin layers and Cooper's ligaments.

The primary application of this technology has so far been in breast imaging as demonstrated by our previous work.^21^, ^25^, ^26^ The performance characteristics described above suggest that the QT scanner can provide high‐resolution, high fidelity, multimodality imaging of the breast tissue. A representative montage of coregistered sets of images of the whole breast are shown in Figs. 12 and 13. In any given image slice, the speed of sound image shows the fibroglandular tissue (shown as higher speed of sound region) embedded within the fat (shown as lower speed of sound region). The skin also has a relatively high speed of sound and can be clearly demarcated throughout the images. The reflection images also highlight skin well since it marks the interface between water and breast tissue and is a source of high specular reflection. Other tissue elements, such has Cooper's ligaments (connective tissue) are also well‐visualized in the reflection image.^21^, ^25^ A magnified view of example speed of sound and reflection images is shown in Fig. 14. The higher speed of sound regions represent terminal ducto‐lobular units (TDLUs) embedded within relatively lower speed glandular tissue.^18^ The fibroglandular region has pockets of and is surrounded by fatty tissue which exhibits relatively low speed of sound. The QT reflection images show remarkable ability in detection of interfaces — Fig. 14(b) shows that QT reflection can differentiate between dermis and epidermis layers of the skin. We would like to point out that the skin region in Fig. 14(b) is better visualized in comparison to Fig. 13 because the contrast (window/level) was optimized for viewing the skin rather than viewing the whole breast. In addition, the posterior breast surface is relatively more perpendicular to the direction of wave propagation resulting in improved visualization of the skin bilayer. As previously shown in our work, the information in the speed and reflection images is highly complementary and using it together provides highly synergistic value to QT's ability to identify breast anatomy.^18^, ^22^

**Figure 15:**
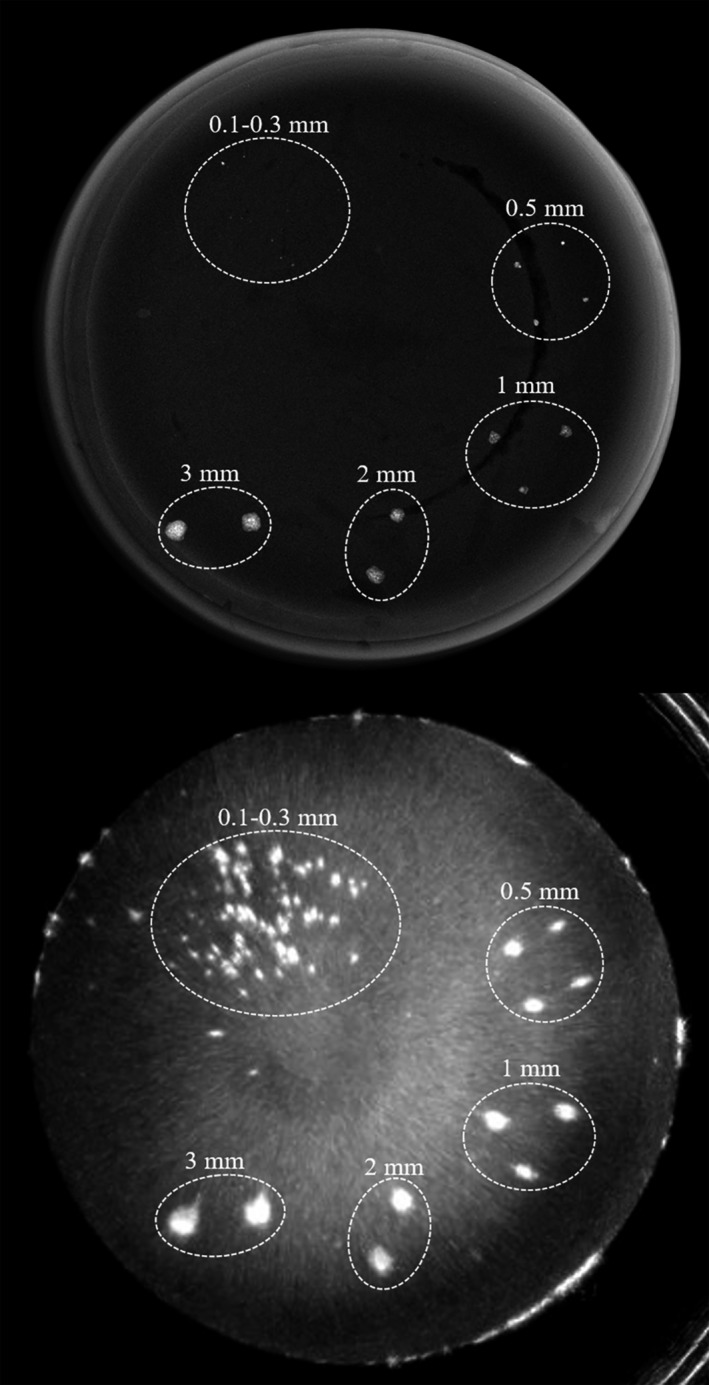
Comparison of mammography with QT reflection images. X‐ray image (top) taken on a mammography system shows calcium particles which can also be visualized in the QT reflection image (bottom).

We further demonstrated the ability of QT reflection imaging to detect microcalcifications in a phantom. Fig. 15 below shows images of an agar phantom acquired on mammography system and on the QT scanner. Note that calcium particles as small as few hundred micrometers can be easily seen on the QT reflection images. Since these particles are below the system resolution and there is “blooming” effect associated with the reflection images, they appear larger than the actual size; however, the contrast is significantly enhanced when visualizing the microcalcifications with QT reflections images.

We are working on improving the quality of the attenuation images which are generated as part of transmission imaging. The attenuation image reconstruction is a more challenging problem to solve due to its ill‐conditioned nature compared to that speed of sound reconstruction.^23^ We are also working on demonstrating the ability of QT ultrasound to detect and isolate calcifications in clinical breast images, which is considered a marker of both prognostic and diagnostic significance.^27^

## 4 Conclusion

We have characterized the imaging performance of QT Scanner 2000. The set of test measurements include characterizing spatial resolution, linear measurement accuracy, and contrast to noise ratio for both speed of sound and reflection modalities, and speed of sound accuracy and image uniformity as well. Clinical images demonstrate the utility of the scanner in breast imaging and show that the scanner is appropriately suited for both research and clinical applications of QT Ultrasound.

## Author Contributions

B.M. and M.L. designed the study; B.M. and R.T. collected and analyzed the data with assistance from M.L.; B.M. wrote the manuscript with assistance from R.T., J.W., and M.L. All authors reviewed and approved the final manuscript.

## Conflicts of Interest

All authors are employees of QT Ultrasound.

## References

[1] Wild JJ , Reid JM . Application of echo‐ranging techniques to the determination of structure of biological tissues. Science. 1952;115:226–230. 14913217 10.1126/science.115.2983.226

[2] Greenleaf JF , Johnson SA , Lee SL , Hermant GT , Woo EH . Algebraic reconstruction of spatial distributions of acoustic absorption within tissue from their two‐dimensional acoustic projections In: Green PS, eds. Acoustical Holography: Volume 5. New York: Springer US; 1974:591–603.

[3] Greenleaf JF . Computerized tomography with ultrasound. Proc IEEE. 1983;71:330–337.

[4] Glover GH . Computerized time‐of flight ultrasonic tomography for breast examination. Ultrasound Med Biol. 1977;3:117–127. 595207 10.1016/0301-5629(77)90064-3

[5] Carson PL , Oughton TV , Hendee WR , Ahuja AS . Imaging soft tissue through bone with ultrasound transmission tomography by reconstruction. Med Phys. 1977;4:302–309. 882064 10.1118/1.594318

[6] Wiskin JW , Johnson SA , Borup DT , Berggren M , Eidens R . Full inverse scattering vs. Born‐like approximation for imaging in a stratified ocean; 1993: pp. III450‐III455, vol. 453.

[7] Wiskin JW , Borup DT , Johnson SA . Inverse scattering from arbitrary two‐dimensional objects in stratified environments via a Green's operator. J Acoust Soc Am. 1997;102:853–864.

[8] Duric N , Littrup P , Babkin A , et al. Development of ultrasound tomography for breast imaging: technical assessment. Med Phys. 2005;32:1375–1386. 15984689 10.1118/1.1897463

[9] Wang K , Matthews T , Anis F , Li C , Duric N , Anastasio MA . Waveform inversion with source encoding for breast sound speed reconstruction in ultrasound computed tomography. IEEE Trans Ultrason Ferroelectr Freq Control. 2015;62:475–493. 25768816 10.1109/TUFFC.2014.006788 PMC5087608

[10] Carson P , Meyer C , Scherzinger A , Oughton T . Breast imaging in coronal planes with simultaneous pulse echo and transmission ultrasound. Science. 1981;214:1141–1143. 7302585 10.1126/science.7302585

[11] Brandenburger GH , Klepper JR , Miller JB , Synder DL . Effects of anisotropy in the ultrasonic attenuation of tissue on computed tomography. Ultrason Imaging. 1981;3:113–143. 7196105 10.1177/016173468100300201

[12] Li C , Duric N , Littrup P , Huang L . In vivo breast sound‐speed imaging with ultrasound tomography. Ultrasound Med Biol. 2009;35:1615–1628. 19647920 10.1016/j.ultrasmedbio.2009.05.011 PMC3915527

[13] Duric N . WE‐G‐210‐00: advances in breast ultrasound imaging. Med Phys. 2015;42:3699–3699.

[14] Sandhu GY , Li C , Roy O , Schmidt S , Duric N . Frequency domain ultrasound waveform tomography: breast imaging using a ring transducer. Phys Med Biol. 2015;60:5381–5398. 26110909 10.1088/0031-9155/60/14/5381 PMC4902020

[15] Ruiter NV , Hopp T , Zapf M , Kretzek E , Gemmeke H . Analysis of patient movement during 3D USCT data acquisition; 2016:979009–979009‐979006.

[16] Lavarello R , Oelze M . A study on the reconstruction of moderate contrast targets using the distorted born iterative method. IEEE Trans Ultrason Ferroelectr Freq Control. 2008;55:112–124. 18334318 10.1109/TUFFC.2008.621

[17] Lavarello RJ , Oelze ML . Tomographic reconstruction of three‐dimensional volumes using the distorted born iterative method. IEEE Trans Med Imaging. 2009;28:1643–1653. 19574162 10.1109/TMI.2009.2026274

[18] Andre M , Wiskin J , Borup D , Johnson S , Ojeda‐Fournier H , Olson L . Quantitative volumetric breast imaging with 3D inverse scatter computed tomography. 2012 Conf Proc IEEE Eng Med Biol Soc; 2012:1110‐1113. 10.1109/EMBC.2012.6346129 23366090

[19] Wiskin J , Borup DT , Johnson SA , Berggren M . Non‐linear inverse scattering: high resolution quantitative breast tissue tomography. J Acoust Soc Am. 2012;131:3802–3813. 22559356 10.1121/1.3699240 PMC3356315

[20] Wiskin J , Borup D , Iuanow E , Klock J , Lenox M . 3D nonlinear acoustic inverse scattering: algorithm and quantitative results. IEEE Trans Ultrason Ferroelectr Freq Control. 2017;64:1161–1174. 28541199 10.1109/TUFFC.2017.2706189 PMC6214813

[21] Malik B , Klock J , Wiskin J , Lenox M . Objective breast tissue image classification using quantitative transmission ultrasound tomography. Scientific Reports; 2016, 6, pp. 38857. 10.1038/srep38857 PMC5146962 27934955

[22] Lenox MW , Wiskin J , Lewis MA , Darrouzet S , Borup D , Hsieh S . Imaging performance of quantitative transmission ultrasound. Int J Biomed Imaging. 2015;2015:454028. 26604918 10.1155/2015/454028 PMC4641199

[23] Wiskin J , Borup DT , Johnson SA , Berggren M , Abbott T , Hanover R . Full‐wave, non‐linear, inverse scattering In: André MP, Akiyama I, Andre M, et al., eds. Acoustical Imaging. Netherlands: Springer; 2007:183–193.

[24] Goerner FL , Duong T , Stafford RJ , Clarke GD . A comparison of five standard methods for evaluating image intensity uniformity in partially parallel imaging MRI. Med Phys. 2013;40:082302. 23927345 10.1118/1.4816306 PMC3745492

[25] Klock JC , Iuanow E , Malik B , Obuchowski NA , Wiskin J , Lenox M . ‘Anatomy‐correlated breast imaging and visual grading analysis using quantitative transmission ultrasound™. Int J Biomed Imaging. 2016;2016:9. 10.1155/2016/7570406 PMC5056280 27752261

[26] Iuanow E , Smith K , Ouchowski N , Bullen J , Klock J . Accuracy of cyst vs. solid diagnosis in the breast using quantitative transmission (QT) ultrasound; 2016. 10.1016/j.acra.2017.03.024 PMC5557662 28549870

[27] Morgan MP , Cooke MM , McCarthy GM . Microcalcifications associated with breast cancer: an epiphenomenon or biologically significant feature of selected tumors? J Mammary Gland Biol Neoplasia. 2005;10(2):181–187. 16025224 10.1007/s10911-005-5400-6

